# The Interplay of Tumor Stroma and Translational Factors in Endometrial Cancer

**DOI:** 10.3390/cancers12082074

**Published:** 2020-07-27

**Authors:** Monika Sobočan, Maria Anna Smolle, Christoph Schatz, Johannes Haybaeck

**Affiliations:** 1 Department of Pharmacology, Faculty of Medicine, University of Maribor, 2000 Maribor, Slovenia; 2 Division of Gynecology and Perinatology, University Medical Centre Maribor, 2000 Maribor, Slovenia; 3 Department of Obstetrics and Gynecology, Faculty of Medicine, University of Maribor, 2000 Maribor, Slovenia; 4 Department of Orthopaedics and Trauma, Medical University of Graz, 8010 Graz, Austria; maria.smolle@medunigraz.at; 5 Area 2 Cancer, Center for Biomarker Research in Medicine, 8010 Graz, Austria; Johannes.Haybaeck@i-med.ac.at; 6 Department of Pathology, Neuropathology and Molecular Pathology, Medical University of Innsbruck, 6020 Innsbruck, Austria; Christoph.Schatz@student.uibk.ac.at; 7 Department of Zoology, Leopold-Franzens University, 6020 Innsbruck, Austria; 8 Diagnostic & Research Center for Molecular Biomedicine, Institute of Pathology, Medical University of Graz, 8010 Graz, Austria

**Keywords:** endometrial cancer, eukaryotic initiation factors, estrogen receptors, epithelial mesenchymal transition

## Abstract

Endometrial cancer (EC) is a common gynecologic malignancy which continues to have a poor prognosis in advanced stages due to current therapeutic limitations. A significant mechanism of chemoresistance in EC has been shown to also be the enhancement of epithelial to mesenchymal transition (EMT) and the subsequent obtainment of stem cell-like characteristics of EC. Current evidence on EMT in EC however fails to explain the relationship leading to an EMT signaling enhancement. Our review therefore focuses on understanding eukaryotic translation initiation factors (eIFs) as key regulators of the translational process in enhancing EMT and subsequently impacting higher chemoresistance of EC. We identified pathways connected to the development of a microenvironment for EMT, inducers of the process specifically related to estrogen receptors as well as their interplay with eIFs. In the future, investigation elucidating the translational biology of EC in EMT may therefore focus on the signaling between protein kinase RNA-like ER kinase (PERK) and eIF2alpha as well as eIF3B.

## 1. Introduction

Endometrial cancer (EC) is the most common gynecological cancer [1,2]. Most ECs are diagnosed in an early stage due to a presentation with symptoms such as postmenopausal bleeding; thus having a good prognosis [2]. Based on their biological potential we currently classify EC into type I and type II EC. Type I EC are endometrioid EC and type II are non-endometrioid types such as clear cell; serous or carcinosarcoma subtypes [3]. However, up to 20% of EC progress to high-stage carcinoma [2].

Besides the tumour itself, its surrounding stroma has gained attention over the last years. The tumor stroma consists of nonmalignant cells specific to each tissue environment as well as immune cells (innate and adaptive), vasculature with endothelial cells, pericytes and the extracellular matrix (ECM) [4]. In order to allow cancers to progress, the microenvironment of the tumor surroundings also needs to change and adapt. Continuous communication develops through this dynamic environment in which cancer growth is promoted [5,6]. Research looking into the understanding of normal endometrial stroma showed that in an in vitro model of Ishikawa cells, the stromal cells regulate growth, differentiation and hormonal responses [7]. Of note, to allow for any biological model to participate in the process of cancer development, at least some of the model components need to be modified. Aberrant processes impacting viability, cell metabolism or protein biosynthesis can all be part of the process of carcinogenesis. While much is known about the changes of the canonical translation process related to cancer development, little is understood of the changes to the physiological process of ribosome recruitment, scanning, initiation, elongation and termination of translation [8].

## 2. The Molecular Landscape of Endometrial Cancer

There have been important advances in the understanding of the molecular landscape of EC. According to the histological profile, type I EC has been associated with unopposed estrogen stimulation. The evolution of type I EC tumors from atypical endometrial cells is connected with PTEN, PIK3CA and CTNNB1 mutations [9]. Studies have also suggested that the presence of an inactivated ARID1A tumor suppressor gene can, together with a PTEN mutation, lead to EC proliferation [9]. In type II serous EC, tumors arise independent of estrogen, but some mutations such as PBXW7, PIK3CA, PPR2R1A mutations and CCNE1 amplifications have been identified as early events in EC progression [9]. Further on, uterine carcinosarcomas (UCS) have coexisting PTEN and TP53 mutations present. This is a characteristic probably due to the nature of the cancer, which is biphasic and has carcinoma and sarcoma characteristics. UCS has also a highly variable EMT transcriptomic gene signature, which could be connected with the poor prognosis of women [9]. Interconnected with the mutational landscape of EC, different signaling pathways are connected with EC progression. These are the pathways: (i) HIF-1alpha, (ii) PI3K/AKT/mTOR, (iii) Ras/Raf/MEK/ERK, (iv) Wnt/beta-catenin, (v) Insulin/Insulin growth factor-1 (IGF-1) [10].

## 3. Eukaryotic Initiation Factors in Carcinogenesis

Eukaryotic translation initiation factors (eIFs) are the key regulators of mRNA translation, composed of the four steps, initiation, elongation, termination, and recycling [11]. With initiation of translation constituting the limiting step, deregulation of molecular processes at this stage have major effects on protein expression [11]. In principle, translation initiation consists of two steps, i.e., the formation of the multifactorial 43S preinitiation complex, and the assembly of the eIF4F complex on the mRNA cap [12]. The preinitiation complex is built up by the 40S ribosomal subunit and associated eIF1, eIF1A, eIF3, eIF5, as well as a complex consisting of eIF2, the initiating methionyl-tRNA, and guanosine triphosphate (GTP), thus also referred to as ternary complex eIF2-GTP-Met-tRNA_i_) [13,14]. The eIF4F complex consists of eIF4A, a DEAD box RNA helicase catalyzing the ATP-dependent unwinding of RNA duplexes [15], the cap-binding subunit eIF4E, and the scaffolding protein eIF4G [13]. eIF4A subsequently unwinds the secondary structure of the 5′ untranslated region (5′UTR) of mRNA after binding of eIF4E to the cap and its connection with eIF4B and eIF4H [13]. The 43S preinitiation complex is then recruited by eIF4G through interaction with three eIF3 subunits (eIF3c, eIF3d, eIF3e) [13,16,17]. The translational processes are frequently disturbed in human malignancies, with several links being present between tumour suppressor genes as PTEN and TP53, as well as oncogenes as MYC and PIK3CA [11,13,18]. Consequently, both overexpression and decreased expression of various eIFs have been linked to enhanced tumour growth, higher grade of malignancy, and worse patient outcome in several tumour entities [19,20,21,22]. Even more, one single eIF may be overexpressed in one tumour entity and underexpressed in another tumour, with examples provided below. This means eIFs may have an oncogenic or tumour suppressive function.

In EC, overexpression of eIF4E is seen in over 50% to over 60% of specimens [23,24]. Moreover, eIF4E-expression correlates with advanced cancer stage, lymph node metastasis and poor patient survival [23,24]. According to Shi et al., expression levels of eIF4E are positively correlated with expression of matrix metalloproteinase 9 (MMP9) in EC [24]. Of note, experimental downregulation of eIF4E leads to a significantly reduced growth of HEC-1A cells, underscoring the important role of eIF4E in EC progression [23].

Related to this, Zhang et al. discovered that micro RNAs (miRNAs) miR-320a and miR-340-5p are downregulated in EC in comparison to adjacent normal tissue [25]. As these two miRNAs bind to the 5′UTR of eIF4E mRNA, they potentially downregulate eIF4E expression [25]. Correspondingly, experimental overexpression of miR-320a and miR-340-5p suppresses migration and invasion of HEC-1A cells. Subsequently, the downregulation of EIF4E and phosphorylated eIF4E results in reduced expression of MMP-9 and MMP-3, thus impairing cellular migration [25]. Transforming growth factor beta 1 (TGF-beta1) is likewise suppressed by overexpression of miR-340-5p and miR-320a, usually being responsible for phosphorylation of eIF4E [25]. Due to the downregulation of TGF-beta1, features of epithelial-to-mesenchymal transition (EMT), normally induced by TGF-beta1, are attenuated in EC tissues in vitro [25,26]. Another eIF, namely eIF2beta, is overexpressed in EC according to the Human Protein Atlas, with high eIF2beta levels being associated with significantly poorer patient survival [27].

Other eIFs aberrantly expressed in EC in comparison to healthy endometrium are eIF2alpha, eIF3C, eIF3H, eIF4G, eIF5, and eIF6 [22]. Whilst eIF6 is downregulated in EC in comparison to healthy endometrium, eIF5 is upregulated [22]. Moreover, expression levels of eIF4G, eIF5 and eIF6 are significantly different depending on EC grade [22]. Of note, eIF4G expression levels are not only significantly higher in type II EC in comparison to type I, but are also independently associated with a worse patient outcome [22].

## 4. Clinical Trials on eIFs in Cancer

Currently, 11 clinical trials are registered on clinicaltrials.gov (retrieval date: 13.07.20), investigating the role of eIFs in cancer, mostly analyzing the effect of agents targeting eIFs (Table 1). Nine of these studies involve agents targeting eIF4E, being either ribavirin, an agent initially developed against hepatitis C [28], ISIS EIF4E Rx, an antisense oligonucleotide against eIF4E [29], or LY2275796, another antisense oligonucleotide blocking the expression of eIF4E [30]. Furthermore, one study analyzed the effect of Zotatifin, an inhibitor of eIF4A1-mediated translation [31]. One study identified aimed at analyzing the amount of phosphorylated eIF2alpha in the tissue of patients with prostate cancer after having received n-3 polyunsaturated fatty acids. However, this trial had been terminated due to slow accrual (ClinicialTrials.gov-identifier: NCT00458549). Two of the trials on eIF4E-targeting agents have been completed and results already published [32,33]. At retrieval date (13.07.20), a further three studies have been completed without results published, one study terminated due to overlap with another ribavirin-investigating trial, two were actively recruiting, one was active but not recruiting, and in one study, the current status was unknown.

## 5. Epithelial Mesenchymal Transition in Endometrial Cancers

EMT is a process in which epithelial cells acquire mesenchymal traits. This is a phenomenon that occurs under physiological conditions and during carcinogenesis [34]. There are three types of EMT. Type 1 is EMT present in the process of embryogenesis and organ development, type 2 is inflammation and fibrosis and then type 3 EMT is the transformation of epithelial cells into cancer cells [10,34]. The type 3 EMT is the one that is connected with the local invasiveness and distant metastasis of cancer and thus is the focus of an ongoing pursuit to understand carcinogenesis [10]. The process of EMT leads to complex changes in the microenvironment such as the loss of epithelial markers (e.g., E-cadherin and beta-catenin) and the upregulation of mesenchymal markers (e.g., N-cadherin and vimentin) [35]. The upregulation of specific genes leads to protein production involved in cell-cell contact, interactions with the ECM and the cytoskeleton changes. These changes are regulated by gene upregulation of EMT enhancers or inducers. Especially the transcription factors of the Twist, Snail and Zeb families have been identified as being significantly involved in this process [36]. The prerequisites for EMT is the remodeling of ECM. The ECM remodeling is directed by the secretion of matrix proteases and scaffolding proteins. The proteins involved are collagens, fibronectin (FN1), plasminogen activator inhibitor 1 (PAI1) and periostin (POSTN) [37]. Especially in EC, research has shown the presence of EMT in more than 60% of EC patients [38].

Genes involved in EMT in EC have been identified through different in vitro studies. By silencing zinc finger E-box-binding homeobox (ZEB1) it was shown that cell sensitivity to cisplatin was improved and EC cell migration decreased [39]. It was shown that ZEB1 interacts with hepatoma-derived growth factor (HDGF) and that by directly suppressing HDGF regardless of ZEB1 status, levels of beta-catenin and TCF4 were decreased thus providing an understanding that ZEB1 as well as HDGF collaborate in EMT transformation in EC [39]. Recently, HDGF was then shown to promote EMT through the activation of AKT-MAPK, Akt and the TGF-beta pathway. Of note, HDGF interacts with the DEAD-box RNA helicase (DDX5) hypothesized to be a coactivator of the estrogen and androgen receptor as well as tumor suppressor p53, MyoD and beta-catenin. Data derived from modelling DDX5 and HDGF showed that PI3K/AKT might regulate beta-catenin through the interaction of the transcriptional factors signaling in Akt and GSK3-beta [40].

When exploring the role of genes connected to estrogen dependent receptors, dual-specificity phosphatase 6 (DUSP6) plays an important role. This gene has been found to dephosphorylate phosphor-tyrosine and phosphor-threonine residues on ERK 1/2 and thus inactivate ERK kinase [41]. ERK 1/2 is considered to be a classic mitogen activated protein kinase which is shown to influence the FGF2 growth factor also overexpressed in EC [42]. DUSP6 studies showed that by knockdown, EMT was enhanced. An overexpression of DUSP6 in endometrial adenocarcinoma led to the formation of polarized cell spheroids and the decrease of cancer cell mobility. This was also seen in increased levels of N-cadherin and decreased levels of E-cadherin [41]. Interestingly, however, Zhang et al. [42] reported that in estrogen dependent cell lines (Ishikawa cells), 17beta-estradiol induced DUSP6 expression and FGF2 expression. However, the mechanisms of this regulation remain to be understood. Estrogen receptors also play an important role in the induction of the ubiquitin-conjugating enzyme E2C (UBE2C) which was shown to be clinically connected to advanced histologic grade, FIGO stage, recurrence and shorter survival. UBE2C knockdown lead to inhibited cell proliferation, migration, invasion and EMT. Knockdown was also connected to an increase of p53 expression [43]. Mutations in the expression of p53 were then further on together with mutations in the PI3K/PTEN and FBXW7 (previously known as CDC4) pathways connected to initiate through EMT also transformation in uterine carcinosarcoma (UCS), a hormonally independent cancer type [44]. Investigating structurally similar receptors as estrogen receptors, Yoriki et al. reported that estrogen-related receptor alpha (ERRalpha) cooperated with peroxisome proliferator-activated receptor gamma coactivator-1-alpha (PGC-1alpha) to induce—together with upregulated EMT markers vimentin, Snail and ZEB-1—the TGF-beta pathway, thus initiating further EMT in EC [45].

The involvement of the gene B-cell-specific Moloney murine leukemia virus integration site 1 (Bmi1) in the carcinogenesis of different cancers has been shown to be correlated with high grade, advanced stage, LN involvement and poor prognosis. Bmi1 helps tumor cells escape apoptotic cell death and has therefore been connected to reduced chemosensitivity [46]. By silencing Bmi1, studies in EC cell lines discovered that expression of SOX2 and Oct4, being transcription factors for totipotency, decreased and the expression of E-cadherin an epithelial marker increased [47]. Highly aggressive forms of EC were also characterized to have increased levels of enhancer of zeste homolog 2 (EZH2). EZH2 serves as a histone methyltransferase that mediates gene silencing on histone H3. In EC it was shown that EZH2 inhibition showed an overexpression of E-cadherin and a decrease of EMT markers [48]. Another transcription factor with an unclear role in EMT in EC is sex-determining region Y-box 17 (SOX17), which was identified as an antagonist and inhibitor of Wnt signaling and a suppressor of beta-catenin activity. Zhou et al. showed that while in a small percentage of EC SOX17 is increased, the presence of an increase was associated with EC metastasis. In in vitro studies this was then shown to be connected with increased beta-catenin expression and decreased E-cadherin levels and as such influencing cell migration [49].

The interconnection of the microenvironment in which EMT develops has also been connected to high glucose environments. It was shown that the dynamin-related protein 1 (Drp-1) increased activation, if there was a high glucose environment present. This is a protein which is key for mitochondrial division. Drp-1 interacted with cells and increased EMT, migration and invasion [38]. By investigating the impact on stem cell activity with metformin, it was further discovered that an adipocyte conditioned media selectively increased stem cell activity. Although Kitson et al. [50] showed that metformin reduced the activity of cells in Ishikawa and Hec-1 cell lines, but not their viability, this effect could not be reproduced using metformin in adipocyte conditioned media. This suggested the importance of investigating the mechanisms of high glucose and adipocyte-conditioned EMT signaling for therapeutic responses in the future. This is additionally supported by reports of the development of spontaneous tumorigenic hybrids between adipose derived stromal cells and EC cells. These fusion processes lead to the increase of cancer cell mobility and migration with an EMT factor upregulation of vimentin and downregulation of E-cadherin [51].

During development of EMT in EC, specific microRNAs (miRNAs), being single stranded small noncoding RNAs of 18–25 nucleotides, play an important role. MicroRNAs were found to regulate body fat [52] and thus have been shown to also correlate with elevated estrogen levels and thus might play an important role in the pathogenesis of EC [53,54]. There are few miRNAs which have been connected to EMT in EC, the identified miRNAs are depicted in Table 2.

The current investigation in EC related EMT regarding gene expression and miRNA expression shows little overlap. The most commonly identified candidate genes were SOX, which has been connected to the Wnt signaling and beta-catenin activity and TWIST, which is a common transcription factor, induced by the cascades of mTOR/PI3K/Akt, MAPK and Rho GTPases [36].

Emerging in our understanding of the biology of cancer is also the role of long noncoding RNA (lncRNA). LncRNAs have long been believed to be transcriptional noise, but are currently emerging as important stakeholders in understanding cancer biology [61]. A particularly important lncRNA for EC seems to be H19, which is a transcript of the gene H19. This lncRNA is highly expressed in fetal tissue and extra-embryonically but is not physiologically expressed in adult human tissue. It has been discovered to be involved in endometrioid EC progression, yet the function was not clear. Using in vitro studies of small interfering RNAs showed a significant decrease of migration and invasion of EC but did not impact growth. lncRNA H19 knockdown lead to Snail downregulation and E-cadherin level increase without affecting vimentin. Thus, there was a partial EMT reversal by knockdown of lncRNA H19 [62]. Emerging as a marker of invasiveness in EC is also the steroid receptor activator (SRA), a lncRNA upregulated in EC compared to normal tissue. Decreasing the expression of SRA decreased the expression of eIF4E-BP and consequently decreasing beta-catenin expression [63].

There is however not always the opportunity to clearly define the origin of tumors with traditional molecular classifications in advanced cancerous lesions. In order to apply with precision based on the tumor biology a type of therapy one must therefore improve the testing of tumors based on their clonality. In EC this becomes especially important once we assess advanced tumors with potential metastasis in the ovary (or the presence of synchronous tumors of the ovary and endometrium) [64]. One possibility for how to overcome this dilemma and better characterize the cancerous lesions is mitochondrial DNA (mtDNA). Mitochondrial DNA (mtDNA) mutations are found in various cancer types [65,66,67,68]. Occurrences are found in every cell stage and a high number of copies are discovered in tumors, suggesting a less tight control and common mutations [69].

Guerra et al. [70] analyzed mtDNA from endometrial cancer, lymph node neoplastic tissue and ovarian cancer and discovered a frameshift deletion m.11038delA in the MTND4 gene in ovarian cancer only. A germ line mutation (m.G15077A) was found in MTCYTB in endometrial cancer, in lymph node and in ovarian cancer [70]. Some 56% (28 of 50) of frozen samples of primary endometrial carcinoma obtained from surgery carried one or more somatic mtDNA mutations, only occurring in the tumor. The D-loop was most frequently affected, and the predominant type of mutation were mitochondrial microsatellite instabilities (mtMSI) [71]. In the 12S rRNA gene either novel mtMSI were found carrying a deletion or insertion of one cytosine residue in front of the thymine or a germline T to C polymorphism was discovered, producing a homopolymorphic C tract resulting in a DNA instability [71]. The base substitution 16189T to C from np 16,024 to 576 have many cancers in common including endometrial, cervical and ovary cancer [72]. Some 32% (16 out of 50) of samples showed changes in the length of a homopolymorphic cytosine at np 303–309 [71]. Within the 7S DNA binding site at the 3′ end and the termination-associated sequence, a CCCCCTCCCC mtMSI was found, leading to an uninterrupted C tract unstable in the tumor [71]. Xu et al. found hypervariable regions within the 16sRNA gene, tRNA(leu) and the ND1 gene associated with a higher risk of developing endometrial cancer [73]. The first data available on mtDNA in EC shows a specific pattern to further explore within the relation it has to EMT and eIF as it is potentially very important in advanced EC.

## 6. Eukaryotic Initiation Factors in EMT

Eukaryotic initiation factors are known to be associated with the prognosis of patients suffering from various cancers. However, little is known on their molecular interactions in regard to EMT. Table 3 represents an overview of different initiation factors and research in connection with eIFs and EC.

**Figure 1:**
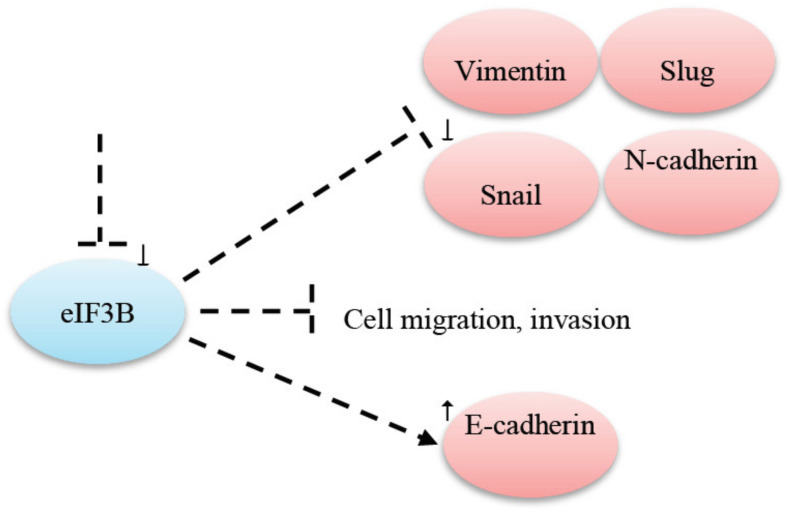
A knockdown of eIF3B prevented cell migration and invasion, decreased the expression of N-cadherin, Snail, Slug and Vimentin and increased the expression of E-cadherin [74].

It is still unclear how eIF complexes work in EMT, with only a few reports existing connecting eIF to EMT. Wang et al. [74] analyzed the impact of eIFB3 complex on gastric cancer. They discovered that eIF3B knockdown prevented cell migration and invasion. It reduced the expression of markers such as N-cadherin, Snail, Slug and Vimentin as well as promoted expression of the epithelial marker E-cadherin [74]. Focusing on eiF3B in EC cell lines, Min et al. found that the downregulation of eIF3B lead to reduced cell proliferation, migration and invasion by silencing the beta-catenin signaling [75]. A proposed model of impact eIF3B overexpression has on EMT factors is represented in Figure 1.

Similarly, studies focusing on eIF5A-2 discovered that this factor was through in vitro studies directly involved in EMT. Through knockdown of eIF5A-2, E-cadherin expression increased and vimentin decreased, this process was reversed, when eIF5A-2 was overexpressed [76].

**Figure 2:**
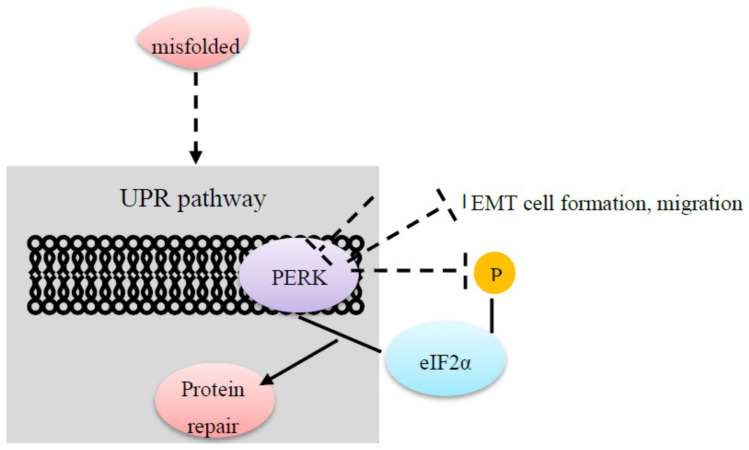
Misfolded proteins can activate the unfolded protein response (UPR) pathway. PERK which is located in the endoplasmatic reticulum membrane interacts with eIF2alpha to induce an integrated stress response to repair misfolded proteins. An inhibition of PERK impacts the phosphorylation of eIF2alpha and leads to a decrease in the EMT cell formation and migration [37].

Factors promoting EMT impact the control pathway of cells and can also impact the unfolded protein response (UPR) which is activated by misfolded proteins. Signaling is impacted by receptors localized in the endoplasmic reticulum membrane. One of the involved receptors in the process is the protein kinase RNA-like ER kinase (PERK) which interacts with eIF2alpha to induce an integrated stress response (Figure 2). This stress response is then connected with selective protein repair. Inhibition of PERK impacted also eIF2alpha phosphorylation in EMT cells [37]. Furthermore, selective PERK inhibition led to reduced EMT cell formation of tumorspheres and migration, but not proliferation. Thus, Feng et al. concluded that EMT supports the selective activation of the PERK- eIF2alpha axis which contributes to noncancerous processes as well as carcinogenesis [37].

This is important as cells in which EMT initiated transformation are understood to have indistinguishable properties in comparison to cancer stem cells (CSC) [77]. Especially in EC, these characteristics have a special place as the endometrial stroma is rich in endometrial mesenchymal stem cells (EMSC) that can regenerate the endometrium and stroma monthly due to the physiological changes happening [78]. The communication between EC cells and EMSC then supports increased levels of TGF-beta1 and C-X-C motif chemokine ligand 12 (CXCL12) expression which subsequently supports increasing cancer cell mobility, invasion and EMT [78]. The controversial role of CSC is activated through different signaling pathways such as Wnt/beta-catenin, Notch1 and Hedgehog signaling [79] involving many of the before mentioned transcription factors.

## 7. Clinical Implications of EMT in EC

Understanding the mechanisms of EMT in EC through their translational biology is important especially with regards to future studies and development of therapeutic agents in patients resistant to first-line chemotherapy. The PERK-eIF2alpha axis seems to be significantly decreased in drug-resistant EC patients. As increased expression of these proteins was shown to be correlated with restricting cell growth and apoptosis, this opens possibilities of treatment of drug-resistant EC. As further investigation by Xu and colleagues [80] shows, EC cells resistant to first line therapy can be sensitized using the knowledge of upregulating proteins communicating with the endoplasmic reticulum membrane [80]. Promising data in in vitro studies also discovered that eIF3B could be an important link to decrease chemotherapy resistance. This is especially seen by the understanding that eIF3B downregulation inhibits EC through inhibiting beta-catenin [75]. Many of the identified transcription factors involved in EMT in EC however have yet to be evaluated in light of our improved understanding of translational biology and EMT markers. Therefore, patient selection refinement to improve therapeutics needs further investigation in the interactions between the main pathways and in the process of EC EMT identified eIFs.

## 8. Conclusions

Cells undergoing EMT show stem cell-like properties and possibly contribute to the reduction of chemosensitivity in patients with EC. Understanding the signaling and factors involved in the process allows us to better select therapeutic targets and candidate patients for considering targeted therapy. Although interactions between different factors have been investigated in an isolated setting, there is still much to be understood about the role aberrantly expressed eIFs play in EC with regards to EMT enhancement. This understanding will potentially provide us with improved biomarkers as well as therapeutic agents to treat EC.

## Figures and Tables

**Table 1:** Clinical trials registered on ClinicalTrials.gov investigating the role of eukaryotic translation initiation factors (eIFs) in cancer *.

ClinicalTrials.Gov Identifier	Tumour	Drug	Purpose	Outcome
NCT01056757	Breast cancer	Ribavirin (eIF4E-inhibitor)	Inclusion of patients with high eIF4E expression	Terminated (overlap with other ribavirin-study).
NCT01309490	Malignant solid tumours	Ribavirin (eIF4E-inhibitor)	Inclusion of patients with high eIF4E expression	Recruiting
NCT01234025	Prostate cancer (castration resistant)	ISIS EIF4E Rx (Antisense oligonucleotide against eIF4E)	Progression free survival following treatment with docetaxel and prednisolone, with/without ISI EIF4E Rx	Completed (no results posted)
NCT01234038	Non-small cell lung cancer (stage IV)	ISIS EIF4E Rx (Antisense oligonucleotide against eIF4E)	Progression free survival following treatment with carboplatin and paclitaxel, with/without ISI EIF4E Rx	Completed (no results posted)
NCT00458549	Prostate cancer	Omega-3 fatty acids	Determine whether neoadjuvant n-3 polyunsaturated fatty acids induce eIF2alpha-phosphorylation in prostate cancer patients	Terminated (slow accrual)
NCT00903708	Advanced cancers	LY2275796 (antisense anti-cancer drug targeting eIF4E)	Pharmacokinetic and pharmacodynamic evaluation of intravenous LY2275796	Completed (no results posted)
NCT04092673	Advanced solid tumours	Zotatifin (= eFT226; inhibitor of eIF4A1-mediated translation)	Dose escalation and cohort-expansion study	Recruiting
NCT01268579	Tonsil and/or base of tongue squamous cell carcinoma	Ribavirin (eIF4E-inhibitor)	Explore whether 2-week ribavirin therapy decreases tumour expression of eIF4E	Active, not recruiting
NCT01675128	Advanced solid tumours, colorectal cancer	ISIS EIF4E Rx (Antisense oligonucleotide against eIF4E)	Pharmacokinetics and pharmacodynamics; investigate maximum tolerated dose and safety of ISIS EIF4E Rx in combination with irinotecan	Completed (Results published [32])
NCT01056523	Acute myeloid leukaemia	Ribavirin (eIF4E-inhibitor)	Pharmacokinetics, pharmacodynamics and efficacy of ribavirin in combination with cytarabine arabinoside	Completed (Results published [33])
NCT02073838	Acute myeloid leukaemia	Ribavirin (eIF4E-inhibitor)	Effect of ribavirin in combination with hedgehog-inhibitor vismodegib and/or cytidine analogue decitabine	Unknown (last status: recruiting)

* Retrieval date 13/07/2020.

**Table 2:** MicroRNAs connected to epithelial to mesenchymal transition in endometrial cancer (EC).

MiR	Target Gene	Function
183 [55]	CPEB1	CPEB1 overexpression mediated 3′-UTR shortening and induces EMT proliferation, migration, invasion.
183 [56]	Ezrin	Upregulation of miR-183 represses Ezrin and reduces metastatic potential.
29 [57]	TPX2	TPX2 overexpression enhances cell proliferation and invasion as well as enhancing apoptosis.
215 [58]	LEFTY2	Upregulation of miR-215 lead to LEFTY2 decrease and subsequent diminishment of mesenchymal to epithelial transition.
326 [54]	TWIST1	TWIST1 and miR-326 are directly correlated and miR-326 is downregulated in EC, leading to cell proliferation, migration, invasion and EMT.
195 [59]	GPER	Overexpression leads to downregulation of MMP-2 and MMP-9 and decreased phosphorylation of PI3K and AKT.
148 [60]	DNMT1	Loss of expression in exomes leads to induction of EMT in CAFs.
194 [53]	SOX3	Upregulation of miR-194 leads to SOX3 suppression and decrease of stem cell invasion.

**Table 3:** The eIFs connected to EC.

Eukaryotic Initiation Factors	Function
eIF3B	eIF3B knockdown prevented cell migration and invasion in gastric cancer [74].
eIF2A-2	eiF2A-2 has a direct involvement in the E-M transition. A knockdown led to an increase in E-cadherin expression and to a decrease in vimentin expression [76].
eIF2alpha	PERK in the endoplasmic reticulum interacts with eIF2alpha for an integrated stress response to repair proteins. Inhibition of PERK impacts EIF2alpha phosphorylation [37]. PERK-eIF2alpha is involved in noncancerous processes and in carcinogenesis [37].
eIF2beta	eIF2beta was found overexpressed in EC [25,26] and is related to a poorer patient survival [27].
eIF3C, eIF3H	eIF3C and eIF3H as well as eIF2alpha, eIF4G, eIF5 and eIF6 were found aberrantly expressed in EC [22].
eIF4E	miR-320a and miR-340-5p were downregulated in normal tissue [25]. eIF4E mRNA is regulated by miR-320a and miR-340-5p [25]. A downregulation respectively a phosphorylation of eIF4E led to an impairment of cellular migration affecting MMP-9 and MMP-3 [25]. TGF-beta1 is responsible for the phosphorylation of eIF4E and was suppressed by miR-320a and miR-340-5p and therefore resulted in an impairment of epithelial-to-mesenchymal transition [25]. eIF4E was found overexpressed in 50–60% of specimens [23,24]. A correlation with advanced cancer stage, lymph node metastasis and poor patient survival was found [23,24].
eIF4G	eIF4G levels were found higher in type II EC compared to type I and led to a worse patient outcome [22].
eIF6	eIF6 was found downregulated in EC [22].
eIF5	eIF5 was found upregulated in EC [22].
